# Placebo and Nocebo Effects Across Symptoms: From Pain to Fatigue, Dyspnea, Nausea, and Itch

**DOI:** 10.3389/fpsyt.2019.00470

**Published:** 2019-07-02

**Authors:** Fabian Wolters, Kaya J. Peerdeman, Andrea W.M. Evers

**Affiliations:** ^1^Health, Medical and Neuropsychology Unit, Institute of Psychology, Faculty of Social and Behavioral Sciences, Leiden University, Leiden, Netherlands; ^2^Leiden Institute for Brain and Cognition, Leiden University, Leiden, Netherlands; ^3^Department of Psychiatry, Leiden University Medical Center, Leiden, Netherlands

**Keywords:** placebo and nocebo effects, suggestion, conditioning, fatigue, dyspnea, nausea, itch, pain

## Abstract

Placebo and nocebo effects are, respectively, the helpful and harmful treatment effects that do not arise from active treatment components. These effects have thus far been researched most often in pain. It is not yet clear to what extent these findings from pain can be generalized to other somatic symptoms. This review investigates placebo and nocebo effects in four other highly prevalent symptoms: dyspnea, fatigue, nausea, and itch. The role of learning mechanisms (verbal suggestions, conditioning) in placebo and nocebo effects on various outcomes (self-reported, behavioral, and physiological) of these different somatic symptoms is explored. A search of experimental studies indicated that, as in pain, the combination of verbal suggestion and conditioning is generally more effective than suggestion alone for evoking placebo and nocebo effects. However, conditioning appears more and verbal suggestions less relevant in symptoms other than pain, with the exception of placebo effects on fatigue and nocebo effects on itch. Physiological measures, such as heart rate, lung function, or gastric activity, are rarely affected even when self-reported symptoms are. Neurobiological correlates are rarely investigated, and few commonalities appear across symptoms. Expectations generally predict placebo and nocebo effects for dyspnea and itch but seem less involved in fatigue and nausea. Individual characteristics do not consistently predict placebo or nocebo effects across symptoms or studies. In sum, many conclusions deriving from placebo and nocebo pain studies do appear to apply to other somatic symptoms, but a number of important differences exist. Understanding what type of learning mechanisms for which symptom are most likely to trigger placebo and nocebo effects is crucial for generalizing knowledge for research and therapies across symptoms and can help clinicians to optimize placebo effects in practice.

## Introduction

The placebo effect, the positive treatment outcomes that cannot be ascribed to active treatment components, has evolved from a nuisance in clinical trials to a phenomenon worth studying in its own right. Placebo effects can influence clinical outcomes in a meaningful way (1) and, under optimal conditions, achieve a large magnitude (2, 3). Moreover, placebo effects occur not just when a placebo is given, but can potentially enhance any active treatment that a patient receives (1, 4). Medical outcomes can be further influenced by the nocebo effect, where, instead of the positive effect in the case of placebo, harmful treatment side effects are evoked or increased, or positive treatment effects are reduced (5, 6).

Most of what we know about placebo and nocebo effects—their magnitude, their working mechanisms, their physiological and neurological correlates—comes from the study of these effects in pain. There are good reasons for this, as pain is well studied, is the most commonly reported somatic symptom (7), and can greatly influence quality of life (8). Pain also has the advantage that it is relatively easy to manipulate and control in laboratory settings: it can be tuned “up” and “down” by exposing the participant to different levels of a noxious stimulus such as heat, cold, or pressure. By contrast, other somatic sensations generally take more time to evoke (e.g., fatigue) or tend to last for a time even after the stimulus is removed (e.g., itch and nausea). This has led to a strong research tradition of studies on placebo analgesia and nocebo hyperalgesia to emerge in the last century, using the benefit of cumulative findings in comparable research settings to thoroughly investigate underlying mechanisms of these effects.

Placebo and nocebo effects play a role not just in pain, but in a wide range of conditions and symptoms. The available research indicates that the underlying mechanisms for these effects might differ per symptom (9). Accordingly, similar procedures might lead to very different results when symptoms are very different, such as pain and hormone levels (10), or to more comparable results when symptoms are more alike, such as pain and itch (11). Symptoms can differ on aspects such as conscious accessibility, the amount of cognitive control one can exert over it, what physiological systems they are connected to, and the related conditions and possible pathophysiological pathways [see, e.g., Ref. (12) for a comparison of itch and pain]. All of these factors can influence a symptom’s susceptibility to placebo and nocebo effects or to learning mechanisms that cause them. The dominant position of pain in placebo and nocebo studies might give the impression that placebo and nocebo effects are only impactful for pain, or that they operate in other symptoms exactly as they do in pain. More importantly, knowing which findings generalize from pain to other symptoms could lead to more effective use of placebo and nocebo effects in both research and clinical practice.

While placebo and nocebo studies of symptoms other than pain are not as plentiful, some lines of research have a long history—for example, the placebo effect was studied in weightlifters and asthmatics in the early 1970s (13, 14). However, as in pain, these studies tend to focus only on a single symptom; there is very little comparative work that examines the similarities and differences between placebo and nocebo effects on pain and these other symptoms. The current review aims to help fill that gap. To facilitate the comparison with pain, we will focus on symptoms that share the features of being subjective, somatic, and commonly reported in the general population (7, 15–17) and that have been studied in the area of placebo and nocebo effects: fatigue, dyspnea, nausea, and itch. We will focus primarily on whether the learning mechanisms that have been established for pain function similarly for these other symptoms, with the status of research for each symptom separately being featured in the discussion. The focus will be on verbal suggestion and conditioning, as other learning mechanisms (such as observational learning) are rarely investigated in the included symptoms [although see Ref. (18) for an exception]. We will first see whether these learning mechanisms are similarly effective at inducing placebo and nocebo effects on fatigue, dyspnea, nausea, and itch as they are at affecting pain. After discussing these results, we will compare the selected symptoms with pain in terms of possible underlying mechanisms, specifically the role of expectations, and individual predictors of placebo and nocebo responses.

## Search Strategy and Selection Criteria

We searched the scientific literature for experimental research on placebo and nocebo effects on subjective, somatic, and commonly reported symptoms other than pain. The included symptoms (and related search terms) were as follows: fatigue (mental fatigue, muscle fatigue), itch (pruritus, antipruritic), nausea (motion sickness, emetic, antiemetic), dizziness (vertigo, fainting), and dyspnea (asthma). These terms were entered in databases PubMed, PsycINFO, and Web of Science in combination with search terms for placebo and nocebo effects (placebo effect, placebo effects, nocebo, conditioning, operant conditioning, classical conditioning, verbal suggestion). Only those studies were included that either mentioned at least one of the included symptoms in the suggestion given to participants or included at least one of these symptoms as a self-reported outcome after a learning procedure featuring verbal suggestion or conditioning. Both studies that included healthy participants and those drawing participants from clinical samples were included. Only experimental laboratory studies were considered, since there are many possible reasons for symptom change in clinical trials and it is unclear whether the change in the placebo group (placebo *response*) is actually due to the placebo (placebo *effect*) or due to other factors such as natural history [see, e.g., Ref. 4)]. This process resulted in no relevant studies for dizziness, and thus this symptom was not further considered. Further studies were added by examining included studies for references and upon expert recommendation.

To answer the question whether expectations play a role in placebo and nocebo effects in fatigue, dyspnea, nausea, and itch, those studies that have explicitly examined participants’ expectations were considered. To examine the question of which traits identify the placebo responder, the studies included based on the aforementioned criteria were scanned for individual characteristics used in moderation analyses. Only variables measured through questionnaires and gender were identified; no studies investigating, e.g., genetic factors were found. A brief summary of the results of included studies can be found in **Table 1**, while a detailed overview of every study is available as **Supplemental Material**.

**Table 1:** Overview of results of included studies.

Learning mechanism	Symptom	Type of measure	Proportion positive results
Placebo—verbal suggestion	Fatigue	Self-reported	7/13
		Behavioral	10/11
		Physiological	1/5
	Dyspnea	Self-reported	3/5
		Behavioral	-
		Physiological	1/5
	Nausea	Self-reported	3/6
		Behavioral	0/2
		Physiological	0/4
	Itch	Self-reported	1/6
		Behavioral	-
		Physiological	0/6
Placebo—conditioning	Fatigue	Self-reported	1/2
		Behavioral	2/2
		Physiological	-
	Dyspnea	Self-reported	-
		Behavioral	-
		Physiological	-
	Nausea	Self-reported	6/7
		Behavioral	1/2
		Physiological	1/4
	Itch	Self-reported	2/2
		Behavioral	0/1
		Physiological	-
Nocebo—verbal suggestion	Fatigue	Self-reported	1/3
		Behavioral	1/2
		Physiological	0/1
	Dyspnea	Self-reported	3/5
		Behavioral	-
		Physiological	3/5
	Nausea	Self-reported	0/3
		Behavioral	1/1
		Physiological	0/1
	Itch	Self-reported	5/5
		Behavioral	1/1
		Physiological	2/2
Nocebo—conditioning	Fatigue	Self-reported	0/1
		Behavioral	1/1
		Physiological	-
	Dyspnea	Self-reported	8/8
		Behavioral	-
		Physiological	5/8
	Nausea	Self-reported	4/4
		Behavioral	2/2
		Physiological	0/1
	Itch	Self-reported	3/3
		Behavioral	0/1
		Physiological	-

Positive results: studies where a significant effect was found in the direction matching the verbal suggestion or learning procedure.

## Placebo Effects

In the prototypical experimental placebo analgesia study, participants are exposed to a painful stimulus, and then receive an inert treatment (the placebo) that is suggested to be an analgesic. This method is easily converted for use in other symptoms by changing the noxious stimulus; for example, instead of applying heat to induce pain, participants cycle on an ergometer to induce fatigue or sit in a rotation chair to induce nausea. The placebo itself is also adaptable: for instance, instead of an analgesic cream, a cream can be described as antiallergenic or an inert inhaler can be described as a bronchodilator. Some studies do not feature a separate inert medication, but directly suggest a change in the method or substance that induces the noxious sensation. For example, electrical stimulation can be described as very likely or very unlikely to cause itch.

Placebo effects can be evoked by only the verbal suggestion of symptom relief, but also by letting participants experience the reduction in stimulus intensity through a conditioning procedure. Meta-analyses have shown that in experimental studies investigating placebo mechanisms, verbal suggestions alone are on average rather effective at evoking placebo analgesia (3, 19). This analgesic placebo effect tends to be further enhanced when verbal suggestion and conditioning are combined (19–21). Conditioning can also be used to evoke placebo effects by itself, without verbal suggestions (21–24), but this is less often investigated in pain and even rarer in most other symptoms we discuss. Other learning mechanisms, such as letting the participant observe the effect in another person, are also rarely examined. Therefore, we will discuss first the effect of only verbal suggestions, and then all studies using conditioning, either paired with verbal suggestion or used by itself. Within each of these categories, fatigue, dyspnea, nausea, and itch are handled in order.

## Placebo Effects Evoked Through Verbal Suggestions

**Fatigue.** A substantial number of studies have investigated the effect of a verbal suggestion of reduced fatigue or increased performance (25–38). In seven of the 13 studies, participants report a lower sense of fatigue while performing a motor task in the placebo condition than in a control condition (25, 28–30, 32, 33, 35). The lack of an effect in the remaining studies might be due to other factors, such as small sample size [Refs. (26, 38); 9 and 10, respectively] or the generic wording of the suggestion [not specifically directed toward fatigue (31, 34) or suggesting a 50/50 chance of placebo (27)]. Across all of the studies, participants also perform better; all but one study (37) find that participants either produced more power or continued a set performance for longer (26–33, 36, 38). Physiological indications of effort, such as blood lactate or heart rate, are often measured, but are not affected in most studies (28, 29, 33, 34, 38), even when the study found effects on fatigue or performance. A decreased readiness potential using EEG during repeated finger movements might indicate that placebo effects on fatigue and performance are caused by a central action in the preparatory phase of movement (35). Thus, overall, it seems that verbal suggestions are effective at reducing experienced fatigue and improving performance, but this is often not accompanied by the expected changes on physiological measures.

**Dyspnea.** Six studies, all using asthmatic participants, have investigated the placebo effect induced by a verbal suggestion on dyspnea (39–44). Three of the five studies that examined self-reported dyspnea report a decrease in symptoms (39, 42, 43). Participants in these studies first received a suggestion of bronchoconstriction (39, 42) or were denied their normal asthma medication (42, 43) before offering participants the placebo which was suggested to improve their breathing. The two studies that report non-significant findings on self-reported dyspnea (40, 44) tested the placebo without first inducing breathing problems. It is likely that a reduction of dyspnea is only expected or possible when it is clearly present in the first place. None of the studies found an effect on measures of lung function (39, 40, 42, 44), except Kemeny and colleagues (41), who did not examine self-reported dyspnea but found an improvement on airway reactivity after a placebo induction. No behavioral or neurological measures were collected. The tentative conclusion from these limited findings is that an existing feeling of dyspnea can be reduced by a verbal suggestion, but likely without accompanying physiological changes [which are themselves not strongly correlated to subjective asthma symptoms; see, e.g., Ref. (45)].

**Nausea.** Eight studies have examined the effect of suggestion of reduced nausea (46–53). Three studies show placebo effects on nausea experienced during a nausea-inducing activity after verbal suggestions (47, 48, 51), although the effect was limited to women in one study (51) and to men in another (48). A possible reason for the non-significant findings in the other studies (46, 52, 53) is that in these studies, participants were not previously made familiar with the nausea-inducing task, possibly resulting in a low expectation of nausea that cannot be reduced further with placebo. Other studies have shown that suggestions of reduced nausea can reduce the disgust experienced when viewing disgusting stimuli (49, 50). With respect to behavioral outcomes, participants did not tolerate the nauseating stimulus for longer after a verbal suggestion of a ginger treatment, regardless of whether there was an effect on reported symptoms (51, 52). Similarly, no differences between the placebo and control groups were detected with an electrogastrogram in any study (46, 51, 52), except for one (53), where participants who received a suggestion of reduced nausea actually showed more abnormal gastric activity. The two functional magnetic resonance imaging (fMRI) studies (49, 50) indicate an effect of a placebo with the suggestion of nausea reduction, showing decreased activity in the insula (particularly the left) and increased connectivity between the dorsomedial prefrontal cortex and the amygdala. The latter finding is consistent with processes of cognitive reappraisal of aversive stimuli, while the insula is a region associated with disgust and pain perception as well as pain analgesia (54–56). The overall pattern indicates that a placebo effect on nausea after verbal suggestion is found only on self-reported measures in some subgroups and under specific conditions.

**Itch.** Six recent studies have examined the effect of suggestions on itch (11, 34, 57–60). In most studies experienced itch was not successfully reduced (11, 34, 57, 59, 60), although it was in one study (58). All but one of the studies that did not find an effect gave only the suggestion, without a separate placebo (11, 59) or used a nonspecific suggestion (34, 60). Regardless of self-reported itch, none of the studies reported an effect on a physiological measure, be it weal size (58, 59), flare (59), skin temperature (59), heart rate (34), or skin conductance (34). Taken together, producing a placebo effect on itch seems to require more than just a verbal suggestion, with effects appearing only with very convincing suggestions or under specific circumstances.

Overall, placebo effects from a verbal suggestion do not seem to be as generally effective in other symptoms as they are in pain. The many studies showing clear effects on self-reported fatigue that extend to performance measures echo results on placebo analgesia, where verbal suggestion alone seems to be effective at reducing pain (3, 19). However, dyspnea, nausea, and itch were not reduced after a verbal suggestion in many studies, and seem to require certain conditions (such as specific phrasing of the suggestion) to be effective. Physiological correlates such as heart rate, lung function, or weal size show little evidence of being affected for any symptom.

## Placebo Effects Evoked Through Conditioning

In a conditioning procedure in a placebo study, participants can personally experience the beneficial effect of the placebo. This is generally done by modifying the intensity of the presented noxious stimulus, such as lowering the heat of a heat pain stimulus when a placebo cream is applied. While conditioning and suggestion were in the past sometimes seen as competing explanations of placebo effects, more recent perspectives (61, 62) generally consider them complementary, both contributing to the expectations that then influence the experience of noxious sensations. Note that when a study involves conditioning, this is almost always classical conditioning; while some studies into operant conditioning in the context of placebo and nocebo effects exist (63, 64), they are as of yet too rare too draw any general conclusions.

**Fatigue.** The combination of verbal suggestion and conditioning to produce a placebo effect on fatigue has been much less studied than the effect of verbal suggestion alone. Only two studies have adopted the method (31, 36). Both studies also include a group where only verbal suggestion was applied, allowing conclusions about the added effect of combining the methods. No effects on fatigue or perceived exhaustion were found in one study (31), while in the other (36), the self-report measure was only affected in the combined verbal suggestion and conditioning group, with no effect of suggestion alone. In both studies, participants in the combination condition showed larger effects on physical performance than those who only received a placebo and the suggestion. The study by Fiorio et al. (31), using transcranial magnetic stimulation, supports the idea that a placebo procedure influences a central mechanism (35), and extends these findings by suggesting that this results in rapid increases in excitability in the corticospinal system for the specific muscle involved. While low in number, these studies suggest greater placebo effects on fatigue and motor performance when verbal suggestion and conditioning were combined.

**Dyspnea.** We are not aware of studies using a design combining verbal suggestion and conditioning or using conditioning alone that investigated placebo effects on dyspnea.

**Nausea.** Two studies have combined verbal suggestion and conditioning to evoke a placebo effect on nausea (48, 65). Horing et al. (65) found that a combination of suggestion and conditioning was effective in reducing both self-reported nausea symptoms as well as behavioral consequences (how often participants could move their head during the nauseating task and how long they could tolerate the task). No results were found on electrogastrogram measures of digestive tract activity. The other study (48) similarly found that self-reported nausea was reduced after a procedure of suggestion and conditioning, but noted that suggestion seemed to be only effective for men, while conditioning was only effective for women. Both studies found an effect that was more elusive in studies that only used a verbal suggestion, indicating that conditioning may have some added value in reducing nausea, but further research will have to elucidate to what extent and for which groups this applies.

For nausea, there is also a line of research into using conditioning alone, without verbal suggestion, to induce placebo effects. These studies use two strategies. The first is overshadowing, where during a learning phase the nausea-inducing stimulus is associated with a very salient stimulus (e.g., a strong-tasting beverage) which is then not present at test (66–68). Because the nausea is associated with the salient stimulus, the absence of the stimulus may reduce nausea. The other is latent inhibition, where participants are exposed to the environment where the nausea is induced several times before the nausea induction (66, 69, 70). There, the fact that the environment is not just associated with nausea but also with previous neutral experiences will make it less nausea-inducing. These protocols have been used to reduce anticipatory nausea (66–69) and nocebo nausea (70). This seems to be generally effective (67–70), although the study implementing both interventions found no differences between the latent inhibition, overshadowing, combination, and control groups, and there were some indications that the latent inhibition intervention actually increased nausea (66). The one behavioral measure of rotation tolerance was not affected (69). Physiological results are mixed: some findings for heart rate correspond to self-report measures (68), but hormone measures either show no effect (67) or follow the unexpected effect of showing increased symptoms for latent inhibition (66). The results of the conditioning studies look promising in reducing self-reported anticipatory nausea and might stimulate other fields to continue to develop optimized conditioning procedures.

**Itch.** Only two studies specifically investigated the effect of verbal suggestion with conditioning on itch (57, 71). In both cases, the placebo consisted of an electrode, with the suggestion that it modified the intensity of the electrically-induced itch. The first study (57) found that only the combination of verbal suggestion and conditioning reduced self-reported itch, with either method individually not producing significant results. The second study (71) further indicated that this combination could also reverse nocebo effects on itch that had earlier been induced by a similar procedure. This reduced itch, however, did not result in reduced scratching behavior (72). While the evidence is limited, these studies suggest that the combination of verbal suggestion and conditioning is needed to successfully induce placebo effects on itch.

Overall, procedures that combine conditioning and verbal suggestion seem to more reliably induce a placebo effect on fatigue, nausea, and itch than those that use verbal suggestion alone. This aligns with results in pain (20, 21). It should be noted, however, that these results are based on a small number of studies, and more confirmatory work will still need to be done. One possible exception and example is the work in nausea, where a stronger tradition of studies has confirmed the utility of conditioning both with and without a verbal suggestion.

## Nocebo Effects

Whereas placebo effects involve the reduction of noxious symptoms, nocebo effects consist of evoking or enhancing these symptoms. The experimental setup of a nocebo study is generally much like a placebo study, where a noxious agent is applied, and the participant learns through a verbal suggestion or conditioning to experience it differently. Nocebo research is still limited because of its relative novelty and the ethical concerns involved. In pain, studies have shown that nocebo effects require fewer learning trials than placebo effects (73) and are resistant to extinction (74). This would suggest more robust findings for nocebo studies than placebo studies. It has also been suggested that nocebo effects on pain might be more reliably evoked with just a verbal suggestion than placebo effects, making the addition of conditioning less necessary [(20); see also Ref. (2)]. We studied whether these findings also apply to fatigue, dyspnea, nausea, and itch.

## Nocebo Effects Evoked Through Verbal Suggestions

**Fatigue.** Three studies have investigated a nocebo effect on fatigue from verbal suggestion alone (25, 75, 76). One study (25) found that the suggestion of a fatigue-inducing drink increased participants’ rate of exhaustion, but did not decrease their performance or influence cardio-respiratory, muscle, and blood lactate measures, while another found the opposite result, with no effect on rate of exhaustion but a reduction in force output (76). The final study (75) did not find increased fatigue after a nonspecific suggestion of ultrasonic noise. This suggests that nocebo effects from verbal suggestion are possible for fatigue when the suggestion is specific enough, but more evidence is needed.

**Dyspnea.** Five studies, all using asthmatic participants, have investigated the nocebo effect of suggestion on dyspnea (39, 40, 42, 44, 77). The results are relatively equivocal: two studies found an effect on reported symptoms (39, 77) while two others found no effect (40, 44) and another only found an effect in a subgroup of highly nervous participants (42). Similarly mixed results are found for lung measures, with two studies finding an effect (44, 77), which was not confirmed in two other studies (40, 42). One study additionally found an effect on a measure of airway inflammation (40). The evidence for nocebo effects on dyspnea and related lung function measures arising from verbal suggestion alone is thus rather tenuous. The overall results are very mixed and no clear methodological trend seems to explain them.

**Nausea.** There are three studies examining nocebo effects from verbal suggestion on nausea (46, 75, 78). No studies found the hypothesized results, reporting no effect on experienced nausea (75, 78), an effect only in men on motion tolerance (78), and a reversed effect (46), where reported nausea and gastric tachyarrhythmia were actually lower for the nocebo group compared to control. The suggestions used in two of these studies were not optimal however, either referring generally to effects of ultrasonic noise (75) or to a drug that would increase nausea but reduce other symptoms of motion sickness (46). These studies suggest that verbal suggestions are not effective for evoking or worsening nausea, but studies using a more specific or unilateral suggestion may prove to be more effective in the future.

**Itch.** Five studies have investigated nocebo effects from the suggestion of increased itch (11, 79–82). All of them indicate that self-reported itch worsened after the nocebo suggestion, although the results were not consistent for every measure in one study (82). Scratching duration was also increased in one study in the group that received a very negative compared to a more neutral suggestion (81), but this also applied to the group receiving no information, and the difference was only seen in patients. The results on self-reported measures seem to transfer to the associated skin reactions (80), although again not consistently for every measure in one study (82). Napadow and colleagues (79) also performed fMRI analyses, finding increased activity in the dorsolateral prefrontal cortex, caudate, and intraparietal sulcus associated with nocebo itch. These areas responded in similar ways to an actual allergen, suggesting this activation may be specific for itch and not applicable to nocebo effects more generally. The results of these studies indicate that nocebo suggestions can worsen experienced itch, while the evidence is more mixed for behavioral and physiological correlates.

Overall, the studies investigating the effect of only verbal suggestions to induce nocebo effects are less numerous than the corresponding placebo studies, and do not provide enough evidence for solid conclusions. Only in itch are consistent nocebo effects seen, which may be due to the unique qualities of that symptom: itch is known to arise even when it is just talked about or observed in others [contagious itch; see Ref. (83) for a review]. The results for itch seem to most resemble those in pain, where nocebo effects seem easier to evoke than placebo effects. For dyspnea and nausea, the results for placebo and nocebo studies are both mixed, and for fatigue the results for placebo are more consistent than for nocebo effects.

## Nocebo Effects Evoked Through Conditioning

**Fatigue.** We are aware of only a single study that has examined nocebo effects on fatigue elicited by a combination of verbal suggestion and conditioning (76). The results indicate no effect on perceived exhaustion, though this might also be due to a training effect emerging throughout the repeated sessions of the experiment. The procedure did lead to an overall reduction in performance, although the effect was not larger in the condition combining verbal suggestion and conditioning than in the verbal suggestion alone condition.

**Dyspnea.** Eight studies have investigated a nocebo effect on dyspnea using conditioning. Two of them combine verbal suggestion and conditioning [Refs. (84–86); note that the latter two use the same dataset), and six rely on conditioning while offering either no suggestion or a similar suggestion in both conditions (87–92). Two studies (84, 87) offer the participant an inhaler and are thus clearly placebo studies, while the others expose participants to scented air *via* a special breathing apparatus but do not offer a physical treatment that would be universally recognized as a placebo. All studies show an increase in self-reported asthma symptoms after conditioning. However, in some studies this increase in self-reported symptoms applied only under certain conditions [i.e., when the conditioning procedure featured an unpleasant and not a pleasant scent (89, 91, 92)] or on some of the included measures [Ref. (87); only subjective airway obstruction was affected, and not feelings of dyspnea or hyperventilation]. Physiological measures of lung functioning and breathing were not affected in three studies (87, 88, 92) while five other studies found an effect on some of the included physiological outcomes (84, 85, 89–91). Many of these studies use healthy samples (84–86, 88–90, 92); the results do not differ systematically between the results of these studies and those where participants were asthmatics (84, 87) or psychosomatic patients (91). These studies together provide convincing evidence that a conditioning procedure can evoke self-reported symptoms of dyspnea, much more clearly than verbal suggestion alone, although the results remain somewhat inconsistent for physiological measures.

**Nausea.** Four studies have used either conditioning alone (78, 93) or the combination of verbal suggestion and conditioning (70, 94) to induce nocebo effects on nausea. In all cases, self-reported nausea was increased in participants, although one study (78) found the same gender pattern as in placebo nausea, where women responded more strongly than men to the conditioning procedure. The gender pattern also applied when considering how long participants could endure the nausea-inducing rotation. Another study using only conditioning (93) found that participants consumed less of a drink that was associated with the rotation, but no effect on tolerance of rotation and also no effect on two hormonal outcomes. The findings indicate that gender may be an important factor in placebo and nocebo nausea. The combination of verbal suggestion and conditioning seems to be quite effective at influencing nausea, and more effective than suggestion alone, although very limited evidence suggests this might not extend to physiological correlates of nausea.

**Itch.** The three available studies indicate that the combination of verbal suggestion and conditioning is effective for inducing nocebo effects on itch (57, 71, 95). All of the available studies find a nocebo effect on self-reported itch when using a procedure combining suggestion and conditioning, although one follow-up analysis (72) did not find consistent effects on scratching behavior. Moreover, the study by van de Sand et al. (95) used fMRI to investigate neural activity associated with nocebo itch, finding increased activity in the rolandic operculum and increased connectivity between the insula and periaqueductal grey in the nocebo condition. These results do not correspond to those found in the earlier fMRI study into nocebo itch (79), which did not use conditioning and only tested patients. Activity in the operculum is also found in fMRI nocebo hyperalgesia studies (96), but these results do not overlap with imaging studies for other symptoms. While no immediate pattern emerges from behavioral or physiological outcomes, self-reported itch is clearly influenced when conditioning and verbal suggestion are combined.

The low number of studies on nocebo effects that use conditioning makes it hard to draw a conclusion across symptoms that is not pre-emptive. The limited results available would suggest that nocebo effects on dyspnea and nausea are more robust after the combination of verbal suggestion and conditioning than they are after verbal suggestion alone. The combination procedure did not seem to lead to more robust effects in fatigue, where results are limited but appear weak, or in itch, where verbal suggestion alone already produced clear nocebo effects. In this sense, only the results for itch seems to echo those on pain, where it has also been suggested that nocebo effects can be evoked as easily with suggestion alone as with a combination of suggestion and conditioning (20).

## The Mediating Role of Expectancies

A common theoretical view is that placebo and nocebo effects function by means of expectancies: you will feel less pain when you expect to (61, 97, 98). In pain, research has shown that the expectation of analgesia or hyperalgesia is indeed a contributing factor to placebo and nocebo effects [e.g., Refs. (99–100)]. Current theoretical perspectives generally consider verbal suggestion and conditioning complementary forces that together influence the expectations that in turn influence the experience of noxious sensations (61, 62).

**Fatigue.** One study (25) found a strong relation between the expectation of increased or decreased fatigue and increases and decreases in performance. Other studies found an effect of the suggestion on participants’ expectations, but no relationship between expectations and fatigue (34) or performance (37).

**Dyspnea.** De Peuter and colleagues (84) found that participants undergoing a nocebo procedure had higher expectations for asthma symptoms as well as higher asthma symptom ratings; however, expectations were only statistically related for asthmatics and not the whole sample. From another angle, a study that specifically tried to not to instill any expectations in participants also found no effects on dyspnea (101).

**Nausea.** Four studies have directly investigated the role expectations in placebo and nocebo effects on nausea (47, 51, 52, 70), but only one of them (47) found the hypothesized effect, with both expectations of nausea and self-reported nausea lower in the placebo than in the control group. The other studies show discrepancies; either expectancies were affected but nausea was not (52), nausea was effected but expectancies were not (51), or there was a relationship between expectations and nausea for nocebo effects but not for placebo effects (70).

**Itch.** The study by van Laarhoven and colleagues (11) showed a correlation between expected itch and nocebo-induced itch ratings. Another study found increased expectations but no corresponding effect on itch (34), while a third found a relation between positive expectations and reduced symptoms only in the experimental group (59). An investigation of the mechanisms behind placebo and nocebo effects on itch (102) found that placebo responders self-generated fewer itch expectations in a separate task, although corresponding results were not found for nocebo responders.

The available evidence points to differential effects of expectations for every symptom, with stronger evidence for expectations as a mediator in itch and some evidence in dyspnea, but more evidence against a mediating relationship in fatigue and nausea. Further research is needed to elucidate what underlying mechanisms might additionally play a role in placebo and nocebo effects in these symptoms, especially for nausea and fatigue where expectations might not play an important role. Other mechanisms such as attention and fear have been suggested [e.g., Refs. (103, 104)], but have only been investigated infrequently, especially outside of pain.

## Identifying the Placebo Responder

The question whether it is possible to recognize the placebo responder is almost as old as the study of placebo effects itself (105), but consistent findings have been elusive (106). One possible reason for the lack of consistent findings could be that predictors are different for different symptoms. We therefore review the findings from the included studies for each of the discussed symptoms.

**Fatigue.** The only study that investigated outcomes on fatigue as well as individual characteristics (34) found no moderating effect of neuroticism, extraversion, positive or negative affectivity, depression, anxiety, catastrophizing, or body vigilance.

**Dyspnea.** Based on a fear learning model (107), it has been hypothesized that participants high in negative affectivity might respond more strongly to negative suggestions of impaired breathing. This has been examined in several studies, with three finding the expected relationship (42, 87, 88) but four others no relationship (41, 89, 91) or an effect only for one of six subjective breathing measures (84). Suggestibility has also been examined as a possible predictor, with one study finding a relationship (77) that was not confirmed in three other studies (39, 41, 42). Likewise, no effect was found in one study for positivity (41). A final study (91) found no effect for information seeking and a negative effect of a blunting behavioral style.

**Nausea.** Two studies investigated the relationship between a placebo effect and multiple individual characteristics. One found a larger placebo effect for participants with lower scores on general self-efficacy, internal locus of control, generalized self, and mobility of nervous processes (108), while the other found no effect of the same variables as well as no effect of anxiety or optimism (51). As mentioned before, in other studies an effect is seen for gender, with men showing larger effects on placebo and nocebo nausea after suggestion and women showing larger effects after conditioning (48, 78), although one study also found suggestion effective only in women (51). There is some indication that this effect may be due to the gender of the experimenter (52).

**Itch.** Several studies in itch have examined many individual characteristics, but found almost no effects for any of them, regardless of whether they found actual placebo or nocebo effects (11, 34, 57, 71). The variables investigated in these studies are theorized to relate to expectations and include neuroticism, extraversion, positive and negative affectivity, depression, anxiety, catastrophizing, body vigilance, optimism, hope, worrying, impulsivity, self-efficacy, general future expectations, suggestibility, and social desirability characteristics. The only one of these studies to find effects (57) did so only in the group where conditioning and verbal suggestion were combined, which was also the only condition that showed effects. Here, a greater placebo effect was associated with less hope, while greater nocebo effects were associated with less hope and extraversion and more worrying, and negative effect. Another study investigating fewer variables (109) found a positive relationship between a placebo effect and ego resiliency but none with neuroticism. Considering the many variables investigated across these studies, the few observed associations should be interpreted with care.

Taken together, these results suggest that individual characteristics do not consistently predict placebo or nocebo effects on fatigue, dyspnea, nausea, and itch. The search for predictors is inconvenienced by the fact that different studies tend to investigate different variables and many results still need to be replicated. It should also be noted that, compared to some other symptoms, the type of variables under consideration is rather narrow, being almost entirely limited to personality factors. Other placebo and nocebo studies have, for example, found indications of genetic predispositions (110) or neurochemical indicators (9). Since placebo effects seem to be determined by a variety of different factors (social, psychological, neurobiological, genetic), future studies may need to incorporate more sophisticated statistical methods to test the combined effect of several predictors at once in order to identify the placebo responder [for some recent examples in pain, see, e.g., Refs. (111, 112)].

## Discussion

This review investigated to what extent findings from studies on placebo analgesia and nocebo hyperalgesia also apply to fatigue, dyspnea, nausea, and itch. Broad similarities can be observed in that placebo and nocebo effects are evoked for these symptoms in a large proportion of studies using similar methods. Some specific results also appear to be consistent: placebo effects are more likely after a procedure combining conditioning and verbal suggestion than verbal suggestion alone, and there is no clear evidence which individual characteristics predict who will respond to placebo and who will not. Other findings do not clearly confirm those in pain. We find little evidence that verbal suggestion alone can consistently evoke placebo and nocebo effects across symptoms, with the exception of placebo effects on fatigue and nocebo effects on itch. For dyspnea and nausea only, nocebo effects seem to be larger after a combination of verbal suggestion and conditioning than suggestion alone. There is some evidence for a mediating role of expectations in placebo and nocebo effects across symptoms, although to a lesser extent in nausea or fatigue. Altogether, it seems that placebo and nocebo studies on pain provide a reasonable starting point for predicting these effects in other sensations, but a number of differences caution against extrapolating every finding in pain to other symptoms.

Each of the sensations we discuss has been studied in a line of research separated from the others, each with its own strengths and opportunities for further inquiry. Studies that examine fatigue tend to come from the field of sport psychology, and therefore focus on improving athletic performance. This has led to placebo effects being investigated much more than nocebo effects. Performance is generally the primary outcome, with perceived exhaustion as just one extra variable. Participants in these studies are generally physically active individuals, sometimes even professional athletes. These factors obviously limit the generalizability of these findings to medical contexts, where fatigue is a large problem (113); it remains to be seen to what extent the findings apply to patients suffering from chronic or mental fatigue. In the context of improving performance, investigating mechanisms behind the effect is perhaps a secondary concern, and fewer studies examine the effect of conditioning or individual characteristics that predict the response. Several researchers have, however, started the work of performing tightly controlled experimental studies that offer more insight into the exact mechanisms of placebo and nocebo effects on fatigue [e.g., Refs. (31, 76)]. It would be fruitful to extend these toward the clinical domain, especially since two separate trials have already shown patients suffering from fatigue can benefit from a placebo intervention (114, 115).

In dyspnea, there is a strong clinical focus, since the background of many of these studies comes from the study and treatment of asthma and somatic symptom disorders. A large proportion of studies therefore also uses asthmatics as participants. This, in turn, also limits generalizability for some findings, albeit in another direction than in the case of fatigue. Since this field features older studies, it also shows more methodological limitations, such as low participant numbers, nonspecific suggestions, and unclear symptom induction methods that can easily be rectified in new studies. Studies that include both conditioning and verbal suggestion, allowing the effects to be compared, would also be a valuable addition. A perhaps bigger issue is that no research group seems to have focused specifically on placebo and nocebo effects in dyspnea. This has led to a lack of common methods and conceptualizations that would facilitate comparisons within the subdiscipline and to other subdisciplines, despite having one of the longest traditions of placebo research [reaching as far back as the study in Ref. (116)]. This might be helped by systematic review or meta-analysis, of the type that exists for other fields [e.g., Refs. (117, 118)].

A clear experimental tradition exists in placebo and nocebo effects on nausea. The field originated in the study of anticipatory nausea in chemotherapy (68, 119), so it has a clear clinical angle, even though later studies focus on healthy participants. The subdiscipline also sports two dedicated research groups and insightful reviews (118, 119). A strong theoretical foundation in conditioning has not only improved upon mixed initial results (46, 52) but has also led to the development of the latent inhibition and overshadowing paradigms that are, as of yet, rarely applied in the rest of the placebo literature. We echo earlier calls (120) that these results should be replicated and applied to other fields of placebo research. This subdiscipline also offers conclusions that deviate the most from findings in pain, with an increased importance of gender [(48, 51, 78); see also Ref. (121) for a recent nuanced overview] and indications of a reduced role of expectations (51, 52, 70). The latter finding is further confirmed by findings in clinical trials that report similarly inconsistent results when expectations are explicitly investigated (122, 123). Our findings do appear to fit with earlier speculation that the gustatory system may have a special capability for unconscious conditioning (124). Due to its connection with the digestive system, nausea may have more in common with symptoms like hormone levels that are more affected by unconscious conditioning than consciously accessible expectations (10). However, since pain is also affected by implicit conditioning (125), further comparisons are needed to resolve this question.

Most studies of placebo and nocebo effects on itch are comparatively recent. This has allowed the field to benefit from advances in other subdisciplines, and thus the studies cover different methods and avoid some of the limitations of earlier work. Itch studies also feature a large number of individual variables as possible moderators, although no consistent findings have emerged. This may be taken as an indication that individual predictors, at least of the kind that can be measured by questionnaires, might not provide much further insight in experimental studies of placebo and nocebo effects. Several studies in the field have also investigated effects arising from verbal suggestion without a physical placebo (11, 59, 80–82), one of which also forgoes deception (59). These are interesting explorations of alternative applications of placebo effects that can also be considered in other subdisciplines. Although the two fMRI studies of nocebo itch do not report clearly congruous results, this is an important first step in investigating whether there is a common nocebo network across symptoms.

Aside from pain, fatigue, dyspnea, nausea, and itch, many other symptoms exist that can be affected by placebo and nocebo effects. Results indicate effects on variables as varied as sleep quality (126), symptoms of Parkinson disease (10), and depression (9). The current review was limited to sensations that share several important similarities with pain, but the question of generalizability of course applies to other sensations as well. Comparisons between these other symptoms can also answer important questions about underlying mechanisms and predictors that cannot be answered for the included symptoms in this review. For example, the neurological underpinnings of placebo effects in Parkinson disease have been studied more (9) than similar mechanisms in dyspnea or nausea. Similarly, there are studies of genetic predictors of placebo effects on pain (127) and fatigue (115), but the available research does not allow a comparison of genetic predictors between the symptoms included in this review. Comparisons might also be valuable for predictors with clear implications for clinical practice, such as the perceived cost of the placebo (128, 129), the odds of receiving placebo (51, 130), the invasiveness of the placebo (131, 132), other interventions that could enhance the effect of placebos (133), or pre-existing associations that could influence symptom acquisition [e.g., the color red being associated with pain (134)].

In order to translate findings to clinical practice, a comparison must also be made between healthy and patient populations. The included studies mostly use healthy volunteers as participants, but a reasonable number focus on patients. While the number of studies that compare healthy and patient populations is too low for a meaningful analysis, there is some indication that patients show different or stronger results (79, 81, 84). A meta-analysis of placebo-like effects on pain in patients (99) tentatively indicated that effects on chronic pain are smaller than on experimentally induced or acute procedural pain, possibly because of a relatively high number of unsuccessful treatment experiences in chronic pain patients. These same experiences, however, should theoretically increase the likelihood of nocebo effects. This is especially relevant considering multiple theories that argue that certain chronic conditions may be exacerbated by or find their etiology in learning effects (107, 135, 136). These theories focus on sensitization, fear learning, conditioning, and generalization, which all likely play a role in nocebo effects. More research that compares healthy groups to those suffering chronically from the relevant symptoms or investigates the progression of these chronic complaints is sorely needed to indicate how much support there is for these theories. This would allow knowledge of placebo and nocebo effects to be utilized to prevent the development and aid the treatment of chronic conditions, such as by counterconditioning the nocebo effect (71).

Our conclusions are limited by the small number of studies available. More studies are needed for solid conclusions, especially about nocebo effects and the added value of combining verbal suggestions with conditioning. Many of the included studies include methodological limitations, such as a small sample size, the omission of a baseline measure, ineffective induction of noxious sensation, or a less convincing verbal suggestion. The included studies also show a large amount of heterogeneity in terms of the methods they use to induce noxious sensations or evoke placebo and nocebo effects. Lastly, the lack of a systematic approach means the review is not exhaustive.

In conclusion, learning mechanisms of placebo and nocebo effects show large overlap, but also important differences across pain, fatigue, dyspnea, nausea, and itch. Knowledge of these differences can be used to optimally control these effects in experimental and clinical studies and increase placebo and reduce nocebo effects in clinical practice. As the separate subdisciplines for each symptom not only provide different results, but also differ in the amount and type of studies available, this review also highlights future promising research possibilities.

## Author Contributions

FW drafted the manuscript, supported by AE and KP. All authors fully read the final draft and provided their approval.

## Funding

The preparation of this manuscript was supported by an Innovational Research Incentives Scheme (Vici) grant from the Netherlands Organization for Scientific Research (NWO) (Grant No. 453-16-004) and an ERC Consolidator Grant from the European Research Council (ERC) (Grant No. 617700), both granted to AE.

## Conflict of Interest Statement

The authors declare that the research was conducted in the absence of any commercial or financial relationships that could be construed as a potential conflict of interest.
