# Supplementary material for: Placebo and Nocebo Effects Across Symptoms: From Pain to Fatigue, Dyspnea, Nausea, and Itch
Source: Front Psychiatry. 2019 Jul 2;10:470. doi: 10.3389/fpsyt.2019.00470 (PMC6614509; doi:10.3389/fpsyt.2019.00470)
Supplement: Supplementary file 1 [file Table_1.pdf]

Table S1 | Overview of selected studies

This table is supplemental material for the following article: Wolters F, Peerdeman KJ and Evers AWM (2019) Placebo and Nocebo Effects Across Symptoms: From Pain to Fatigue, Dyspnea, Nausea and Itch. Front. Psychiatry 10:470. doi: 10.3389/fpsyt.2019.00470

Author	P/N	N	Population	Sensation evocation	Placebo type	Verbal suggestion	Conditioning procedure	Control condition	Self-report measures	Behavioral/physiological/neurological measures
Fatigue										
Kalasountas et al., 2007	P	42	Healthy, ?%F	Weight lifting, 2 trials 1 day apart	Milk-sugar tablets	Told tablets contained amino acids with immediate strength effects	None	Baseline trials (within). Placebo on first trial but not on second, or no placebo at either	Interview. 56 - 75% expected positive effects, felt invigorated after placebo, felt better between trial days, and disappointed/less effective when placebo was canceled	Increase in max lift weight that disappeared when placebo did or increased further with second placebo
McClung et al., 2007	P	16	Athletes, 33%F	1 km time trial	Drink solution	Told active treatment (sodium bicarbonate) plus additive that would prevent side effects	None	Told additive only*	Lower RPE in the told drug conditions	Lower blood lactate levels in the drug groups, shorter total time in told drug groups
Pollo et al., 2008	P	44	Healthy, 0%F	Leg extensions until exhaustion	Decaffeinated coffee	Presented as strong coffee	Surreptitious weight reduction	Decaffeinated coffee presented as such. Weight as experimental	Lower RPE only in VS+ conditioning group	Higher muscle work in VS group, even higher in VS + conditioning group
Duncan et al., 2009	P	12	Healthy, 0%F	Single-leg leg extensions	Sweetened water	Told caffeine, given literature about the positive effects of caffeine on performance	None	Told placebo and consumed nothing conditions (within)	Higher RPE in told placebo condition compared to control and told caffeine conditions	Higher weight lifted and more repetitions performed in the told caffeine condition compared to other conditions. No effect on cardiovascular measures
Duncan, 2010	P	12	Athletes, moderate caffeine users, 0%F	Wingate test on cycle ergometer	Sweetened water	Told caffeine, given literature about the positive effects of caffeine on performance	None	Told placebo*	Lower RPE in placebo caffeine group compared to the told placebo group	Higher peak power output in placebo caffeine compared to told placebo. No differences in mean power output, fatigue index, peak blood lactate, or peak heart rate
Pollo et al., 2012	N	67	Healthy, 0%F	Leg extensions until exhaustion	Sham electrical stimulation	Told electrical stimulation would increase fatigue	Surreptitious weight increase	No sham stimulation. Weight as experimental	No differences in RPE	Lower muscle work from VS (exp1) and VS + conditioning (exp2), but no difference between VS and VS + conditioning
Bottoms et al., 2014	B	12	Healthy, 0%F	Arm crank until exhaustion	Sugar-free drink	Drinks described as 'sports performance' or 'fatigue inducing'	None	Given water (within)	Lower RPE in placebo and higher RPE in nocebo condition. Higher expectancies in placebo and nocebo groups. Expectancies related to performance	Higher performance in placebo group vs control, no difference between nocebo vs control. No differences on cardio-respiratory, muscle and blood lactate measures.
Fiorio et al., 2014	P	60	Healthy, 48%F	Single finger piston press	Sham TENS	Described as effective treatment for enhancing force production	Surreptitious cursor excursion amplification	No TENS or told TENS in ineffective	Higher expectations and judgement of effectiveness in VS + conditioning group. Higher rating of effectiveness and force expenditure lower in VS group. Only a difference between control groups on RPE.	Higher force in VS + conditioning group and lower in control groups vs baseline. More neural activity in the relevant part of the motor cortex in experimental groups
Peerdeman et al., 2015	P	116	Healthy, 71%F	10 min submaximal bicycle test	Placebo capsule	Told reduced sensitivity to physical sensations, effective in 95% of users	None	"effective in 5% of users"	No differences in intensity or unpleasantness ratings	No differences in heart rate or skin conductance
Piedimonte et al., 2015	P	30	Healthy, right-handed, 47%F	Finger flexation for 30 min	Decaffeinated coffee	Presented as strong coffee	None	No substance given	Lower RPE in placebo group vs control	Lower readiness potential on EEG
Tolusso et al., 2015	P	10	Healthy, 0%F	Running-based anaerobic sprint test on treadmill	Water with "water enhancer"	Brochure on positive research results on the substance	None	Water condition (within)	Lower pain in the placebo condition. No effect on RPE.	Higher peak and mean power in the last set on day 2, but not at any other time. No differences in metabolism
Tallis et al., 2016	P	11	Healthy, 0% F	Knee extend and flex of the dominant leg (40x)	Sweetened water	Told caffeine, given literature about the positive effects of caffeine on performance	None	Told placebo, given caffeine or given caffeine and told placebo (within)	No differences in pain perception. Expectation not or negatively (1 measure) related to performance	No differences in performance
Brietzke et al., 2017	P	9	Healthy, ?%F	Cycling maximal incremental test until exhaustion	Sucrose capsule	Described as caffeine	None	Given actual caffeine or no substance	No differences in RPE	No differences in maximal oxygen update. Higher time to exhaustion and peak power output in placebo and caffeine conditions vs no substance; no differences between placebo and caffeine
Broelz et al., 2018	P	34	Athletes, 0%F	45 min cycle ergometer time trial	Either salient pudding or nonsalient capsules	Told 50/50 chance of placebo or actual ergogenic supplement	None	Given nothing	No differences in RPE	Higher blood lactate in salient placebo group vs control and nonsalient placebo groups. Higher power output in both placebo groups. No difference for believing to be in the placebo or real group
Flectcher et al., 2018	N	40	8 symptomatic (50%F), 32 asymptomatic (47%F)	Sustained attention response task	VS only	Suggestion of ultrasonic sound	None	Actual ultrasonic sound, told unknown if sound, or told no sound	No differences in self-reported fatigue or nausea. Higher ear pain and dizziness in asymptomatic group and tinnitus in symptomatic group	Higher galvanic skin response in US present vs US absent cue only in asymptomatic group
Fanti-Oren et al., 2019	P	24	Children age 9-11 av., 50%F	Treadmill exercise stress test	Water drink	Described as increasing energy level, muscle strength and exercise performance	None	Described as water (within)	Lower average and peak RPE in placebo condition	Higher peak heart rate, higher exercise test stage achieved, longer time to exhaustion and shorter recovery time in placebo condition
Dyspnea										
Isenberg et al., 1992	B	33	Asthmatics, ?%F	3 inhalers, baseline - constrictor - dilator	Placebo inhaler	Described as bronchoconstrictor or bronchodilator ('strong but shortlived' effects)	None	Baseline measure (within)	Higher asthma symptoms after constrictor and lower after dilator	No effect on FEV1 or later measures

Van den Bergh et al., 1995	N	28	Healthy, 50%F	Breathing mask	Scented air with 0% CO2	Told minor transient complaints could appear while breathing both odors	Scent associated with 7.4% CO2	Nonassociated scent (within)	Higher self-reported respiratory complaints after conditioning, but only for the unpleasant scent	Higher breathing frequency after conditioning, but only for the unpleasant scent
Van den Bergh et al., 1997	N	28	Psychosomatic patients, 50%F	Breathing mask	Scented air with 0% CO2	Told minor transient complaints could appear while breathing both odors	Scent associated with 7.4% CO2	Nonassociated scent (within)	Higher self-reported respiratory complaints after conditioning, but only for the unpleasant scent	Higher heart rate and breathing frequency after conditioning
Van den Bergh et al., 1998	N	56	Healthy, 50%F	Breathing mask	Scented air with 0% CO2	Told minor transient complaints could appear while breathing both odors	Scent associated with 7.4% CO2	Nonassociated scent (within)	Higher self-reported respiratory complaints after conditioning, but only for the unpleasant scent	No effect on respiratory responses or heart rate
Van den Bergh et al., 1999	N	64	Healthy, 75%F	Breathing mask	Scented air with 0% CO2	Told minor transient complaints could appear while breathing both odors	Scent associated with 7.4% CO2	Nonassociated scent (within)	Higher self-reported respiratory complaints after conditioning	Higher breathing frequency (but no other respiratory measures) after conditioning, but only for ammonia
Devriese et al., 2000	N	56	Healthy, 59%F	Breathing mask	Scented air with 0% CO2	Told minor transient complaints could appear while breathing both odors	Scent associated with 7.4% CO2	Nonassociated scent (within)	Higher self-reported respiratory complaints after conditioning	No effects on respiratory behavior
Leigh et al., 2003	N	17	Asthmatics, 8 suggestible (75%F) and 9 suggestion-resistant (56%F)	Saline inhaler	Saline inhaler	Told bronchoconstrictor/could cause wheezing/chest tightness (first), told broncholdilator (second)	None	Baseline and actual metacholine challenge	No difference in dyspnea after sham bronchoconstrictor. Correlation between dyspnea increase in sham and metacholine conditions in suggestible but not suggestion-resistant group	5 suggestible vs 1 nonsuggestible participant showed reduced FEV-1 in the constrictor phase. No effects for the dilator phase
Put et al., 2004	B	32	Asthmatics, ?%F	Inhale twice for each inhaler, test - constrictor - dilator	Placebo inhaler	Described as bronchoconstrictor or bronchodilator ('strong but shortlived' effects)	None	Placebo inhaler described as practice/just air (within)	Lower symptoms after bronchodilator. No effect for bronchoconstrictor. No effects for social desirability.	No effect on lung function measures
De Peuter et al., 2005	N	38	50% asthmatics and 50% healthy, 50%F	6 2-minute rebreathing trials + two test trials	Placebo inhaler	Both substances described as possibly having short-lasting effects on airways	Inhaler coupled with high (5%) CO2	Other inhaler coupled with pure oxygen (within)	Higher symptom expectancies and subjective symptom ratings in test phase for CO2-associated inhaler for both groups. Expectancy correlated with symptom scores only in the asthmatic group.	Higher respiratory drive in test trials with CO2-assoicated inhaler for both groups; no difference on other respiratory measures
De Peuter et al., 2007	N	30	Asthmatics, 73%F	Histamine inhalation + test trials with saline	Saline inhaler	None	Histamine provocation	Baseline (within)	Higher ratings of obstruction and fatigue (but not dyspnea) after saline inhaler.	No difference in lung function
Kemeny et al., 2007	P	50	Asthmatics, 56%F	Inhaler before methacholine challenge	Placebo inhaler	"You shouldn't have any symptoms"	None	Baseline (within)	None	Improved airway reactivity (PC20) after placebo vs baseline. No correlations between placebo response and social desirability, positivity, NA, physician attitude
Fannes et al., 2008	N	56	Healthy, 52%F	Breathing mask	Scented room air	Told minor transient complaints could appear while breathing one odor	Scent associated with 20% CO2	Nonassociated scent (within)	None	Higher ventilation that reduced with increased trials
Meulders et al., 2010	N	56	Same as Fannes et al. (2008)	Breathing mask	Scented room air	Told minor transient complaints could appear while breathing one odor	Scent associated with 20% CO2	Nonassociated scent (within)	Higher respiratory complaints only in good predictors for one of the odors	None
Wechsler et al., 2011	P	39	Asthmatics, 80%F	None	Placebo inhaler (blinded) or sham acupuncture	Neutral double blind instructions	None	Active treatment with albuterol and no intervention (within)	Lower asthma symptoms for all intervention conditions vs no intervention. No difference between active treatment and both placebo conditions.	No difference between the two placebo conditions and control on FEV1 . Higher FEV1 in alburterol vs control
Jaén et al., 2014	B	17	Asthmatics, 47%F	Odour in one nostril through olfactometer	Odor only	"some people have reported produced mild respiratory problems" or "some people have described them breathe better"	None	Baseline measure (within)	Higher irritating and annoying scores in harmful odor group vs helpful group. No differences in asthma symptoms	No differences in FEV1, etCO2 and respiratory rate. No differences in heart rate or variability. Increased airway inflammation in the harmful odor group, at 0, 2 and 24 h after exposure

Nausea

Stockhorst et al., 1998	P	16	Cancer patients receiving chemotherapy, 50%F	Chemotherapy infusion	Conditioning only	Conditioning only	Overshadowing with strong-tasting drink	Given water	Lower number of patients reported anticipatory nausea in overshadowing group. Lower duration and higher latency of post-infusion nausea in overshadowing group.	More vagal activity in control vs overshadowing group
Klosterhalfen et al., 2000	N	29	Motion sickness susceptible, 54%F	Rotation chair with vertical head movement	Conditioning only	Conditioning only	Stongly favored drink just before rotation	Water just before rotation	Higher anticipatory nausea in conditioned group. No differences in pre or post rotation nausea	Lower strong-tasting drink consumption in conditioned group. No effect on rotation tolerance or hormone levels
Williamson et al., 2004	B	80	50%F, either low or high motion sickness susceptible	Rotating optokinetic drum or non-moving drum	VS only	Told to either expect motion sickness or pleasant sensations	None	None	No difference between high and low expectation groups in motion sickness	Higher abnormal gastric activity in the low expectation group than the high expectation group
Klosterhalfen et al., 2005	P	24	Healthy, 50%F	Rotation chair with vertical head movement	Conditioning only	Conditioning only	Latent inhibition with pre-exposure to rotation chair, 3 or 1 sessions	No pre-exposure to rotation chair	Lower anticipatory nausea in pre-exposure groups vs control at test. No differences during learning. Higher nausea and more reduction due to latent inhibition in women vs men	No group differences in rotation tolerance on days 3 and 4
Levine et al., 2006	B	75	Healthy, 27%F	Rotating optokinetic drum	Placebo pill	Tested motion sickness medication or experimental motion sickness medication that could enhance nausea	None	Placebo pill described as placebo	Lower nausea in the nocebo group. No difference between placebo and control	Lower gastric tachyarrhythmia (measured by EGG) in the nocebo group during drum rotation. No difference between placebo and control group
Klosterhalfen et al., 2009	N	96	Motion sickness susceptible, 50%F, 48 in each experiment	Rotation chair with vertical head movement	Listerine Cinnamon breath strip	Told stimulus (breath strip) can worsen motion sickness (experiment 2 only)	Given breath strip two times before (experiment 1 only)	Given breath strip hours after rotation (exp 1); no information provided (exp 2)	Anticipatory nausea higher in conditioning group in women in exp 1. Post-rotation nausea higher in conditioning group and even higher in women in exp 1. No differences in nausea in exp 2.	Lower rotation tolerance in women after conditioning in exp 1 and in men after suggestion in exp 2

Weimer et al., 2012	P	64	Healthy, 50%F	Rotation chair with vertical head movement	Placebo pill	Told ginger pill	None	Told placebo*	No differences in nausea symptom ratings. Symptom ratings and expectancy ratings did not affect any other results. Lower expectancies in told ginger conditions	No differences between groups for the number of rotations or head movements or rotation time. No differences in EGG activity
Horing et al., 2013 (experiment 2)	P	32	Motion sickness susceptible, 50%F	Rotation chair with vertical head movement	100 ml water with lemon aroma	Told antiemetic medication (ginger solution)	Surreptitious rotation speed decrease	Told water	Lower maximum nausea symptoms in placebo group	Higher rotation tolerance and higher number of head movements in placebo group. No differences in EGG activity
Schienle et al., 2014	P	34	Right-handed, 50%F, at least average disgust proneness	Watching disgusting pictures in fMRI	Silica capsule	Herbal medicine used to treat digestive problems and fever which would reduce nausea	None	Not given any substance (other day, within)	Lower disgust rating for disgusting pictures in placebo vs control condition	Lower left insula activity in placebo vs control condition; difference correlated with the reduction in disgust ratings. Higher amygdala-DMPFC coactivation in placebo condition
Stockhorst et al., 2014	P	24	Healthy, 50%F	Rotation chair with vertical head movement	Conditioning only	Conditioning only	Overshadowing with strong-tasting beverage	Given water in all sessions	Lower anticipatory nausea in overshadowing group vs control. No difference in post-rotation nausea	No group differences in cortisol and TNF-alpha. Lower cortisol and TNF-alpha in women, but higher in men, in overshadowing group vs control
Hall et al., 2015	P	32	Healthy, 100%F	Rotation chair with vertical head movement	Conditioning only	Conditioning only	Latent inhibition (pre-exposure to rotation setting), overshadowing (strong-tasting beverage) or both	No pre-exposure (early sessions in a neutral office context) and drank water	No difference in anticipatory nausea. Test session nausea higher in latent inhibition group and lower in overshadowing group compared to control, but only when not corrected for baseline	Higher cortisol levels in latent inhibition group vs overshadowing group. No differences for other groups
Quinn et al., 2015	N	30	Healthy, 53%F	Galvanic vestibular stimution	Inactive (monopolar) GVS	Told device may temporarily cause low levels of nausea	Earlier active (bipolar) GVS trials	Earlier trials were also inactive (monopolar)	Higher nausea for the conditioned groups than control. No effect for context change	None
Müller et al., 2016	P	21	Motion sickness susceptible, 100%F	Rotation black and white stripes presentation	Sham TENS accupuncture	Told 50/50 chance active TENS accupucture	None	Natural history condition (within)	Lower nausea increase and nausea expectation in placebo vs natural history group	None
Quinn et al., 2016	P	56	Healthy, 57%F	Galvanic vestibular stimution	Peppermint vapor (either on day 2 for conditioning groups or 3 for VS only group)	Told vapor reduced nausea (on day 2 in the combined group and day 3 in the VS only group)	Low intensity GVS on day 2	Received vapor on day 3 with no instructions	Higher nausea symptoms in both VS and conditioning groups. VS effective for men but not for women, conditioning for women but not for men.	None
Quinn et al., 2017	B	90	Healthy, 53%F	Galvanic verstibular stimulation	Inactive (monopolar) GVS	Told device may temporarily cause low levels of nausea	Earlier active (bipolar) GVS trials or additional pre-exposure with inactive GVS	Earlier trials were also inactive (monopolar)	Higher nausea in conditioned groups. Lower nausea in pre-exposure group. Expectancies related to nausea increase, but not decrease	None
Schienle et al., 2017	P	45	Right-handed, healthy, 100%F	Watching disgusting pictures in fMRI	Silica capsule	Homeopathic medicine able to reduce disgust-related symptoms, effective in previous study	None	Neutral pictures, passive viewing condition (both within)	Lower disgust ratings for disgust pictures in the placebo vs control condition	Lower insula and DLPFC activity in placebo vs control. Lower connectivity between left insula and right amygdala/right insula, lower connectivity between right DLPFC and right amygdala, and higher connectivity between bilateral amygdala and left orbitofrontal cortex in placebo vs control
Weimer et al., 2017	P	48	Healthy, 50% female	Rotation chair with vertical head movement	Colored and flavored water drops	Told antiemetic medication (ginger solution), either 50% or 100% chance	None	Told water; baseline rotation without placebo (within)	Lower symptoms in both VS groups vs control, in women only. No difference between VS groups. No effect of expectations or individual moderators	No group differences in increase in rotation tolerance or decrease in EGG ratio
Flectcher et al., 2018	N	40	8 symptomatic (50%F), 32 asymptomatic (47%F)	Sustained attention response task	VS only	Suggestion of ultrasonic sound	None	Actual ultrasonic sound, told unknown if sound, or told no sound	No differences in fatigue or nausea. Higher ear pain and dizziness in asymptomatic group and tinnitus in symptomatic group	Higher galvanic skin response in US present vs US absent cue only in asymptomatic group
Itch										
Scholz et al. 1994	B	30	AD patients, ?%F	Histamine skin prick	VS only	Instruction that exaggerated the possible skin reaction	None	"instruction that played down the potential reaction"	Higher itch ratings in exaggerated suggestion group than downplayed suggestion group	Higher skin reactions in exaggerated suggestion group than downplayed suggestion group
van Laarhoven et al., 2011	B	105	Healthy,100%F	Mechanical or electrical stimulation	VS only	Told 95% experience itch and 5% experience pain or vv (part 1); told nearly all don't experience itch/pain anymore (part 2)	None	Told hardly anyone experiences pain or itch (part 1) or same instruction as in part 1 (part 2)	Higher itch in nocebo vs control condition. No difference in placebo vs control condition in part 2. Expectancies correlated with nocebo effect	None
Bartels et al., 2014	B	95	Healthy, 77%F	Electrical stimulation	Sham electrode	Told color cue indicated intensity change	Pairing of color cue with different intensity stimulation	No information given, intensity was independent of color cue	Higher symptom scores in nocebo trials and lower in placebo trials vs control trials for VS + conditioning group, but not for VS or conditioning groups	None
Darragh et al., 2015	P	48	Healthy, 79%F	Histamine prick	Inert aqueous cream	Presented as antihistamine treatment cream	None	Presented as control cream (within)	Lower itch in placebo condition at one, three and five minutes but not at seven minutes	No difference in weal size
Napadow et al., 2015	N	14	AD patients, 57%F	Histamine prick with thermal modulation	Water drop	None given, but expected to be histamine due to earlier exposure to procedure	None	Histamine and neutral conditions (within)	Higher itch in nocebo vs neutral. Increases in itch during nocebo trials correlated with levels of placebo-induced itch reduction	Higher activity in DLPFC, caudate, and intraparietal sulcus in nocebo vs neutral

Peerdeman et al., 2015	P	116	Healthy, 71%F	Histamine iontophoresis	Placebo capsule	Told reduced sensitivity to physical sensations, effective in 95% of users	None	"effective in 5% of users"	No differences in intensity or unpleasantness ratings	No differences between groups in heart rate or skin conductance
Schut et al., 2016	N	120	50% AD patients, 50% healthy	Watching itch-inducing video (lecture on crawling insects)	Video only	Told videos would induce itch (informed group) or would definitely cause awful itch (catastrophizing group)	None	No information about upcoming video (between); control video (within)	None	Higher scratching duration in catastrophizing and uninformed groups as compared to informed group, but only in AD patients
Stumpf et al., 2016	N	100	Healthy, 50%F	Histamine prick and sodium chloride prick	VS only	"I'm going to inject you with a small quantity of a substance that causes an enormous itch in most people"	None	Neutral suggestion, more mild suggestion ("some itch")	Higher itch intensity and unpleasantness ratings in nocebo vs control for sodium chloride, but no effect on urge to scratch. Higher itch intensity in nocebo vs control for histamine, but no effect on unpleasantness or urge to scratch	Higher weal, but not flare size in nocebo sodium chloride condition. Higher flare , but not wheal size in nocebo histamine condition
Bartels et al., 2017	B	129	Healthy, 78.7%F	Electrical stimulation	Sham electrode	Told color cue indicated intensity change	Pairing of color cue with different intensity stimulation	Conditioning and extinction groups (phase 2 only)	Higher itch in nocebo condition in phase 1. Effect increased by further conditioning, increased less by extinction, and reduced by counterconditioning in phase 2. Effects generalized to histamine itch in phase 3	Higher scratching behavior in learning trials, but not in test trials, both in phase 1 and phase 2 of the experiment (analysis from Bartels et al., 2018, using data from the same experiment)
Van de Sand et al., 2018	N	30	Healthy, right-handed, 60%F	Histamine patch with thermal modulation	Sham TENS	Told "subluminal TENS" had been shown to aggravate itch by experimenter in white lab coat	Surreptitious increase of histamine concentration and lowering of temperature	No histamine and told no TENS conditions (within)	Higher itch ratings in the nocebo condition vs histamine condition	Itch related to right mid-posterior insula activity; nocebo itch related to activity in the contralateral rolandic operculum and PAG
Meeuwis et al., 2018	P	91	Healthy, 81%F	Histamine iontophoresis	Open-label verbal suggestion	Told 95% of people experience little to no itch with this procedure; told about the effect of suggestion	None	No suggestion	Lower itch expectations in placebo condition, but not itch rating. Expectation related to itch in experimental group only. Higher positive affect in the experimental group, no difference in negative affect	No differences in weal or flare size or skin temperature
Skvortsova et al., 2018	P	108	Healthy, 100%F	Histamine iotophoresis	Nasal spray	Told oxytocin which decreases itch and pain sensitivity	None	No suggestion*	Lower expectations after positive suggestion. No differences in itch intensity	None

Note. P = placebo, N = nocebo, B = both placebo and nocebo, F = female, VS = verbal suggestion, TENS = transcutaneous electric nerve stimulation, RPE = rate of perceived exhaustion, GVS = galvanic vestibular stimulation, EGG = electrogastrogram, FEV1 = forced expiratory volume in 1 second, DLPFC = dorsolateral prefrontal cortex, PAG = periaquaductal grey
*Balanced placebo design study. Apart from placebo and control group, also includes active treatment groups that either received the same suggestion as the placebo group or the same suggestion as the control group.
